# Looking back in time: conducting a cohort study of the long-term effects of treatment of adolescent tall girls with synthetic hormones

**DOI:** 10.1186/1471-2458-11-S5-S7

**Published:** 2011-11-25

**Authors:** Fiona J Bruinsma, Jo-Anne Rayner, Alison J Venn, Priscilla Pyett, George Werther

**Affiliations:** 1 Mother and Child Health Research, La Trobe University, 215 Franklin St, Melbourne, Victoria 3000, Australia; 2 Menzies Research Institute, University of Tasmania, Private Bag 23 Hobart, Tasmania 7001, Australia; 3 School of Population Health, The University of Melbourne, Level 5, 207 Bouverie Street, Carlton, Victoria 3010, Australia; 4 Department of Endocrinology and Diabetes and Centre for Hormone Research, Royal Children's Hospital and Murdoch Childrens Research Institute, Flemington Rd, Parkville, Victoria 3052, Australia

## Abstract

**Objective:**

Public health research is an endeavour that often involves multiple relationships, far-reaching collaborations, divergent expectations and various outcomes. Using the Tall Girls Study as a case study, this paper will present and discuss a number of methodological, ethical and legal challenges that have implications for other public health research.

**Approach:**

The Tall Girls Study was the first study to examine the long-term health and psychosocial effects of oestrogen treatment for tall stature.

**Results:**

In undertaking this study the research team overcame many hurdles: in maintaining collaboration with treating clinicians and with the women they had treated as girls - groups with opposing points of view and different expectations; using private practice medical records to trace women who had been patients up to forty years earlier; and exploring potential legal issues arising from the collection of data related to treatment.

**Conclusion:**

While faced with complex challenges, the Tall Girls Study demonstrated that forward planning, ongoing dialogue between all stakeholders, transparency of processes, and the strict adherence to group-developed protocols were keys to maintaining rigour while undertaking pragmatic research.

**Implications:**

Public health research often occurs within political and social contexts that need to be considered in the planning and conduct of studies. The quality and acceptability of research findings is enhanced when stakeholders are engaged in all aspects of the research process.

## Introduction

Public health research often involves investigation of topics that are broad and complex and generates findings with social as well as health implications. This paper tells the story of the Tall Girls Study and uses it as a case study to consider issues that may arise in other public health research. The paper begins with the background to the Tall Girls Study. It then discusses aspects of study design, particularly in regard to accessing an appropriate cohort, tracing study participants and practicalities of data collection. Ethical issues and consumer participation are also discussed.

The first long-term follow-up of girls treated with oestrogens, the Tall Girls Study, was undertaken in Australia between 2000 and 2003 [1]. The study examined a broad range of health and psychosocial outcomes in women treated with high dose oestrogens as adolescents in an attempt to reduce their adult height and compared them with women who were assessed but not treated for tall stature. While the short-term side-effects of this treatment, including weight gain, menstrual irregularities, nausea, night cramps and limb pains, benign breast disease, excessive vaginal discharge, thrombosis and ovarian cysts were well documented [2-5], nothing was known about the long-term health and psychosocial outcomes of this treatment. The study contributed to a broader understanding of the associations of high levels of oestrogen in adolescence on reproductive [1] and other outcomes [6-9].

## Background

Very little information is available on the evolution of the use of high dose oestrogens as a treatment for tall stature in healthy adolescents. After the successful use of this treatment in children with pituitary gigantism in the 1940s [10], clinicians in the United States (US) extended the treatment to tall girls with concerns about their height but who had no associated pathology [3]. The first report of treatment of tall girls was published in 1956 [11]. There have been no randomised controlled trials of treatment effectiveness, and cohort studies have demonstrated that treatment is only moderately effective [9]. Despite this, the treatment became an accepted clinical practice in many countries including Australia [12,13].

Treatment was based on the knowledge that normally in puberty oestrogen inhibits further increases in height by fusing the epiphyses (growth-plates) of the long bones. Most treating clinicians considered treatment acceptable if a girl’s adult height was predicted to be above the 97^th^ percentile or two standard deviations above the mean female adult height in the given population [2,4]. Determination of predicted adult height involved estimating bone age after an x-ray of the hand and wrist and assessing the remaining growth potential [14-16]. The various methods for calculating predicted adult height are prone to error [17].

The earliest predicted height criterion for oestrogen treatment in the US was 173cm in 1956 [11,18,19] and this gradually increased over time but continued to fluctuate between countries. During the 1970s, Australian girls with a predicted adult height of ≥177cm were eligible for treatment [2]; in the US, girls whose predicted adult height was above 173cm [20] or 182.9cm [21] were also treated; and in Germany and the Netherlands the treatment criterion was a predicted height of ≥180cm [22,23]. Age at initiation and duration of treatment also varied considerably and was strongly associated with overall treatment effectiveness. The clinical challenge was that the accuracy of predicting adult height increased after the age of ten years (or closer to puberty) however the potential height reduction diminished [24]. Some clinicians waited until girls grew to an arbitrary height before initiating treatment: 167cm in Australia [2]; 169.5cm in the US [24]; and 170cm in Europe [25]; while others waited until girls reached puberty when they were more likely to be psychologically ready for the effects of treatment on their development but when treatment may be less effective [26]. Duration of treatment varied but was generally two to four years [2,23,27]. Treatment regimes varied considerably and initially were a combination of oestrogen and testosterone [11]. Various preparations of oral synthetic [2,23,28] or conjugated oestrogens [25] were given continuously or cyclically; oestrogen pellets were inserted under the skin at six monthly intervals [29] for some; and intramuscular injections of oestrogen were given to others [24,26,30,31].

The rationale for treatment was to reduce perceived psychosocial ‘risks’ tall girls may face in adulthood. These included physical deformity, through tall girls’ adoption of slouching in an effort to reduce their height; and psychological distress and social withdrawal as a consequence of tall girls’ difficulty conforming to cultural stereotypes [2,3,18,24,32,33]. However, there was very little reporting of any evidence for these assumptions [13] and there are very few published studies reporting psychosocial outcomes following treatment. Three relevant studies [33-35] were limited by small sample sizes, the participants being still relatively young, and the fact that the investigators were treating clinicians which may have affected responses. While treatment is relatively uncommon now in Australia, it was still being used in the US in 2002 [36]; is currently recommended for the management of familial tall stature [37]; and more controversially has been proposed as a form of clinical management of disabled children to reduce their height and increase manageability [38].

The use of synthetic oestrogens as a medical treatment to reduce the height of tall girls was relatively unknown among the general public during the decades it was at its peak in Australia and elsewhere. Even within some families a girl’s height assessment and treatment was kept a secret from other family members.

Public awareness of the treatment of tall girls came about in Australia in 1997, when the treatment became part of a wider debate in the Australian Federal Parliament on the use of human biological products and post-war medical experimentation [39]. Subsequent to the media coverage of this issue, the need for a comprehensive follow-up study was raised independently both by Australian endocrinologists and by women who had been assessed and treated for tall stature as adolescents. These women formed a lobby group, Tall Girls Inc., to petition the government for a follow-up study citing a range of health concerns and that their families had been unaware of the experimental nature of this treatment [40].

Clinicians and Tall Girls Inc. alike had concerns about the possibility of long-term effects of oestrogen treatment, particularly exposure to diethylstilboestrol (DES), which is associated with serious adverse outcomes when given to pregnant women [41,42].

In early 1998, the paediatric endocrinology group at the Royal Children’s Hospital was under increasing pressure to conduct a follow-up study to examine long-term outcomes of treatment and was looking for a group of researchers with whom to collaborate. Researchers at La Trobe University who were interested in the research and had significant experience in conducting long-term follow-up studies in women’s health met with treating clinicians. Following an agreement for the need for a study and for formal collaboration, a period of intense discussion and consultation was undertaken with all parties to formulate the study design and research questions. Given their investment in the project and the sense of disenfranchisement that many treated tall girls felt from the original decision-making, Tall Girls Inc. and particularly the president, Janet Cregan-Wood, were involved from inception in the study design process. In 2000, the National Health and Medical Research Council (NHMRC) provided funding for the study (known as the Tall Girls Study).

## Study design

The study was, by necessity, designed as a retrospective cohort study. Ideally, the cohort would be comprised of all Australian women assessed for tall stature. However, the number of women who were assessed and/or treated in Australia was not known. What was known was that the private practice of one paediatric endocrinologist comprised a high proportion of treated tall girls. Meticulous records had been kept and the specialist was willing to make them available for the study. In addition, a large number of women had been assessed for treatment as adolescents but their estimated adult height did not warrant treatment or their parents elected not to have the treatment. All treated and untreated (assessed only) women were considered eligible to participate and the inclusion of untreated women provided an important comparison group of tall women who had been through similar referral pathways.

To ensure the most complete coverage possible, a number of direct and indirect strategies were used to maximise recruitment. These included having access to the private medical records of the main treating clinician so we could trace women through public records and invite them to participate in the study; having access to the mailing list of Tall Girls Inc. so we could invite women directly; advertising the study through the mailing list of a tall women’s clothing company; inviting other paediatric endocrinologists who may have treated tall girls to participate; and advertising through professional medical, nursing and ancillary associations. Some tall girls were referred for a height assessment by doctors and nurses undertaking yearly health checks in private girls’ schools and so information about the study was placed in alumni newsletters of these schools.

While these strategies increased the opportunity for women to self-refer into the study, reliance on self-referral as the only recruitment method would have resulted in a study sample that was too small and findings prone to selection bias [43] if volunteers over-represented women reporting adverse outcomes.

A cohort of 1,432 eligible subjects was identified: 1,248 from medical records (1,222 of these from one paediatric endocrinologist) and 184 from self-referrals. 572 women in the cohort were treated and 860 women were untreated. A total of 1243 (90% of treated and 84% of untreated) were traced and invited to participate in the study: of these, 398 treated and 448 untreated women agreed and completed a postal questionnaire. Women were also invited to complete a computer assisted telephone interview (CATI) which collected information on reproductive history and a measure of mental health measure; 371 treated women (72% of those traced) and 409 (56%) untreated women completed this. Written consent to abstract data from medical records was provided by 93% (n=726) of women who completed the interview. However, medical records were available for only 618 (75% of treated and 95% of untreated).

The mean age of participants was 39·8 years (range 20-55) in the treated group and 37·7 years (range 23-54) in the untreated group. Treated and untreated participants were similar in their marital status and highest level of education achieved. Self-reported current height was greater in treated women (mean 179·0cm, SD 4·0cm) than in untreated women (mean 176·8cm, SD 4·9cm). Similarly, the first recorded estimated mature height was greater in the treated women (mean 181·5cm, SD 6·3cm) than in the untreated (mean 174·3cm, SD 4·6cm).

The issues that we encountered in assembling a cohort were very similar (on a smaller scale) to those encountered by researchers aiming to investigate the health outcomes for women and their offspring who were administered diethylstilbestrol (DES) during pregnancy or were exposed in-utero [44], in that it was impossible to enumerate the total exposed cohort, accessing medical records posed a number of challenges and it was difficult to trace women over a long period of time.

### Clinical collaboration

In Australia, the medical records of private medical practitioners are the property of the medical practitioner, not the individual to whom the record refers. Under both the Privacy Act 1988 (Commonwealth) and the Health Records Act 2001 (Victoria), records cannot be divulged to a third party without the individual’s consent, although there are some exemptions, one of which is where the research is in the public interest. It is a well accepted practice that health providers will contact patients on behalf of researchers to invite them to participate in relevant research. If the individual agrees, contact then happens directly between the participant and the research team. In Australia, private practitioners are not obliged to keep records of children once they reach 25 years of age or seven years after last contact.

This provision protects privacy and trust in the doctor-patient relationship however, it also opens the possibility of gate-keeping by clinicians. This issue requires further ethical consideration as a clinician may decide the research is not appropriate, or that the patient would not be interested or that participation would not be in the patient’s best interests, thus removing choice from the individual.

These provisions have implications for investigating and reporting the long-term safety and outcomes of treatment. In the Tall Girls Study, we ascertained through Tall Girls Inc and the Australian Endocrinology Society that at least 51 other Australian doctors provided treatment. The research team traced and sought assistance from 35 doctors (16 were either in nursing homes or deceased) to determine whether they had treated tall girls, still had the girls’ medical records and would be willing to recruit these women to the Tall Girls Study. Thirty-two doctors responded to the request: seven were willing to assist and could identify up to 100 eligible women; eight doctors had retained their medical records but could not readily identify those of tall girls; 12 doctors treated women but their records had been destroyed or were no longer in their possession; two doctors were unwilling to recruit women; and three had no recollection of having treated or assessed girls. Researchers investigating outcomes following use of DES faced similar challenges, with records destroyed, ineligible records or physicians being unwilling to participate [44].

In the case of the Tall Girls study, agreement was made between the private medical practitioners and the research team that the medical practitioners would release a small amount of information to researchers for tracing purposes (with no medical or treatment details) with appropriate human ethics committee approval. Once a current address was obtained, the medical practitioner would contact women on the researchers’ behalf. During the tracing phase of the study, the specialist with the largest number of records died, however his family, in-line with his wishes, allowed the project to continue. Ethical approval was obtained for a modification to the approach in that the letter was sent by the researchers outlining how the woman’s details had been obtained. While this meant that we were directly contacting the majority of women (a small number of whom were uncomfortable about this), it also had benefits. Some women were angry about their treatment and may not have responded to an approach directly from their doctor. The research team took particular care to emphasise that while collaborating with, and in contact with both clinicians and Tall Girls Inc., the research was conducted by an independent research team. In all cases, women were initially contacted by mail using a standardised letter. The opening paragraph incorporated minor modifications according to whether the research team was conducting the woman directly, as a result of a self-referral to the study, the research team were directly conducting women who were former patients of the deceased practitioner or a clinician was sending the letter to a former patient on behalf of the research team.

### Tracing and recruitment

One of the greatest challenges the study faced was tracing women between 14 and 40 years after they were assessed or treated for tall stature. Much of the information from medical records was out of date, as parents were unlikely to still be living at the same address and most women would have changed their surname with marriage. Despite assistance from the Australian Electoral Commission (AEC), who performed record linkage on name and date of birth, tracing women using the medical record data to try and link names and addresses over time involved tedious and time-consuming trawling through years of public records including electoral rolls, telephone books and death notices, all on microfiche. The National Death Index (NDI) was also used to record link registered Australian deaths with the names and dates of birth of women. Tracing and contacting women to invite them into the study was crucial to help minimise selection bias and to build confidence in the study findings. Considerable effort was made in locating women who may have changed their name.

Once again, this experience had many parallels with the experience and strategies used in the Diethylstilbestrol-Adenosis (DESAD) project conducted by Nash and colleagues [44]. The Tall Girls Study traced 87% of women identified as eligible from medical records [1], a great result given the limited information available and the duration of time to follow-up and similar to that traced by Nash et al (1983) [44]. The effectiveness of different strategies will vary greatly between countries; it is important to tailor strategies according to local circumstances to obtain optimal results. For instance, in Australia, voting is compulsory so use of the electoral roll provides good population coverage, where voting is not compulsory this will not be the case.

In some instances, following detailed tracing, researchers were still hesitant about the accuracy of the match. In order to safeguard privacy, and due to the sensitivity of the topic, ethics approval was obtained to send a very generic letter (with no information regarding the research topic) verifying the accuracy of the match. Once the match was verified, the initial recruitment pack was sent. In other instances, an address was located for the parents but not the child. In this case we contacted the parents and asked them for contact details of their daughter(s) or requested that they forward to them our letter of invitation. Some parents were reticent or refused to do this as they felt it would raise issues they would rather let lie. In these instances we tried to collect limited data from the parents on their daughter’s health and relationship status.

In all instances it is vital for rigorous epidemiological research that access to sources for tracing be maintained. Privacy is important; however this must be balanced with the public expectation that information is available on the long-term impact and effects of medical treatments. Studies would be unable to be conducted, or the validity of which would be compromised, if access to named or identifying data on a population-based level was not possible.

The issues outlined in the previous two sections, namely storage and retrieval of medical records, availability of records and willingness of practitioners to participate, tracing and recruitment of participants, have ongoing implications for public health research. Each year new treatments and new medications are added to standard clinical practice, the long-term implications of which cannot always be foreseen [44]. An interesting example, with some parallels to the treatment of tall girls, is the current use of hormone treatments for short stature [45]. Another example is the potential need to trace women administered human pituitary growth hormone (hGH) [46] and human pituitary gonadotrophin [47]. Tracing plays an important part in accurately reporting outcomes and reducing bias in findings. It requires access to population-based identifying data sources or use of a unique identifying number. However, prior to this, accurate records of treatment must be kept.

### Practicalities of data collection

The Tall Girls Study collected data on multiple health and psychosocial outcomes. This necessitated a range of quantitative and qualitative methods each of which had specific challenges. The primary exposure of interest was the synthetic oestrogens used to accelerate puberty and retard growth (height) which would be ascertained through access to medical record data. A postal questionnaire collected self-reported information on current health status using standard validated tools, treatment history, family attitudes to tall stature, perceived advantages and disadvantages to tall stature and satisfaction with treatment using structured and open-ended questions; and personal and demographic details including current height. Information considered more complex or sensitive including reproductive and sexual history and mental health status was collected using a computer assisted telephone interview (CATI). While the majority of women participated in all components of the study, a small number of women chose to only participate in a component of the study. Postal questionnaires were mailed to women with the initial approach letter outlining the study. Return of a completed questionnaire implied informed consent. Included with the postal questionnaire were two separate consent forms, one for permission to access their medical record for assessment and treatment details, and the second for permission to be contacted for a CATI interview.

Due to the number of telephone interviews that needed to be conducted over a relatively short period of time and the difficulties in staffing and scheduling these, it was decided to contract these interviews to a commercial interviewing company. Once interviewing commenced it became clear that the interviewers had very little experience in health, they did not tailor contact to the times that women had already indicated were convenient and although initially they had indicated that they were happy for the project manager to be on site and monitor some of the interviews, in practice this was not the case. The interviews were significantly more complex and took significantly longer than they had estimated in their costing (despite a number of briefings). The commercial company was not willing to address these issues and only completed half of the CATI interviews.

The remaining interviews were conducted in-house by the project manager and two members of the project team. This change meant that any queries about the study or associated issues regarding being an assessed or treated tall girl could be clearly and accurately addressed. An additional advantage of the project team being involved in data collection was that it gave a good sense of the strengths and weaknesses of data items which then informed the analysis process. While conducting the interviews in-house had advantages, it also had costs (both economic and emotional). A small number of CATIs needed to be conducted with women who were living overseas and it required rescheduling of staff work hours as a high proportion of interviews needed to be conducted between 5 and 9pm at night and in some instances in the early hours of the morning. Some of these interviews were also emotionally taxing and the three in-house interviewers conducted between 50-100 interviews each over the space of a few months. Regular debriefing was required; this was conducted by other team members.

Analysis of one of the primary outcomes, fertility, collected by the CATI did not find any significant difference in rates of reporting between the interviews conducted in-house or outsourced, which was reassuring in term of study validity. A difference was found for the outcome depression, with higher rates found by the in-house interviewers and one interviewer in particular. Depression was collected using a standardised instrument, the CIDI. It is not possible to determine how much the differences were in the result of how the instrument was administered as opposed to differences in the characteristics of the women interviewed. More importantly, conducting the interviews in-house had some less quantifiable benefits. For some participants the interview was the first time they had discussed their experience of assessment or treatment with anybody and even though this wasn’t part of the CATI wanted to share this as well as sometimes seeking information about why certain examinations/tests were done. In-house interviewers were able to listen (without commercially driven time constraints) and appropriately respond to their stories or requests for information. This may also have broader long-term effects by providing women with a positive experience of participating in research, perhaps making them more likely to participate in other studies in the future.

This experience illustrates the need for experienced research staff to conduct interviews that are of a sensitive and complex nature. This is important both for ensuring the quality and accuracy of the data collected but also in terms of providing a positive experience of research for participants. The need for staff training and debriefing should not be overlooked.

### Ethical issues

The use of information from private medical records without individual consent for the purposes of research raises important legal and ethics issues [43,48]. As has already been mentioned the study required access to medical records held by private medical practitioners. The research team needed to balance obtaining data on long-term outcomes with not harming participants. For some women, the study brought back difficult memories and raised a topic that had previously been taboo within the family. The research team put together detailed protocols regarding these issues. A small number of women (less than five) were concerned about how their details were obtained and these were immediately reported to the ethics committee and were successfully resolved.

A more contentious issue was access to medical records. Some women had been trying unsuccessfully to access their medical record via the private practitioner and there was previous (and current at the time the study began) legal action between a small number of women and one medical practitioner. At the beginning of the study the research team investigated whether study data could be subpoenaed in litigation or whether data collected for the study could be accessed via a Freedom of Information request. Examination of Victorian freedom of information legislation suggested that it may be possible but legal advice indicated that a request for an exemption could be made given that such a precedent may jeopardise research more generally.

The knowledge that we were able to access the records for research purposes prompted some women to ask us to provide them with the information. Many women could not remember what treatment they had received and were eager to know. This placed the research team in an unusual and difficult position: we had obligations to the medical practitioners who were assisting us with our research, and to study participants [43]. After discussion with representatives of the primary medical practitioner concerned, and after legal advice and in line with the health privacy principles of the Victorian Health Records Act (2001), we developed a policy where women could be provided with a summary of the data that was abstracted from the medical record and also a summary of the data collected using the study instruments. If the woman wanted to see the medical record or obtain copies of photos or other original material she needed to contact the representative of the relevant medical practitioner. In practice, less than 10 women requested access to their study records.

### Consumer collaboration

The involvement of Tall Girls Inc. was crucial for a number of reasons. One reason why the study was being conducted was to address their concerns about the possible adverse outcomes of treatment. Their support of the study was vital in terms of encouraging women to participate and promoting the study through their networks and the media. The topic was particularly emotive for this group of women. However, as any one study is unable to answer all research questions, the research team felt the participation of Tall Girls Inc. in the research design process from the beginning to be important. This involved numerous and lengthy discussions about what the project could and could not do, as well as their input into study questions. While time-consuming, we believe that this investment of time and genuine involvement in the research process had dividends for both the research team and members of Tall Girls Inc. It ensured that the study questions were relevant. At the end of the study the group had more realistic expectations about the findings and accepted them more readily. Some members of Tall Girls Inc reported to the research team that they found their involvement in the process to be empowering. While consumers and clinicians were involved in the development of the study methodology and data collection instruments, the study was conducted independently of both stakeholder groups. Public health investigation intersects frequently with the community and community advocacy groups; a particular example is the investigation of cancer clusters. Very few cancer clusters are found to be attributable to a particular cause or exposure but frequently the findings are received with scepticism. The experience of active engagement and explanation of research process and design may assist in this understanding.

## Implications

This paper has provided the social and historical context of a retrospective cohort study. Our experience with undertaking this study has implications for wider public health research. We believe active engagement with stakeholders provided a positive experience of the research process, improved the design of the study instruments and facilitated acceptance and understanding of the study findings. It also highlights the need for accurate record-keeping and systematic storage of records over an extended period of time and for access to records that allow tracing to take place. Further clarification of ethical and legal access to records would facilitate studies investigating long-term outcomes of interventions and treatments.

## Competing interests

The authors declare that they have no competing interests.

## Authors’ information

AV, GW and PP were responsible for the design, conduct and interpretation of the Tall Girls Study. FB, JR and AV contributed to the design and interpretation of the original study and the conception and writing of the paper.
